# Perioperative and extended outcomes of patients undergoing parastomal hernia repair following cystectomy and ileal conduit

**DOI:** 10.1007/s00345-024-05123-w

**Published:** 2024-08-12

**Authors:** Taseen F. Haque, Alireza Ghoreifi, Farshad Sheybaee Moghaddam, Masatomo Kaneko, David Ginsberg, Rene Sotelo, Inderbir Gill, Mihir Desai, Monish Aron, Anne Schuckman, Siamak Daneshmand, Hooman Djaladat

**Affiliations:** Urology Department, University of Southern California, Los Angeles, CA USA

**Keywords:** Abdominal hernia, Cystectomy, Herniorrhaphy, Surgical mesh, Urinary diversion

## Abstract

**Purpose:**

To report perioperative and long-term postoperative outcomes of cystectomy patients with ileal conduit (IC) urinary diversion undergoing parastomal hernia (PSH) repair.

**Method:**

We reviewed patients who underwent cystectomy and IC diversion between 2003 and 2022 in our center. Baseline variables, including surgical approach of PSH repair and repair technique, were captured. Multivariable Cox regressionanalysis was performed to test for the associations between different variables and PSH recurrence.

**Results:**

Thirty-six patients with a median (IQR) age of 79 (73–82) years were included. The median time between cystectomy and PSH repair was 30 (14–49) months. Most PSH repairs (32/36, 89%) were performed electively, while 4 were due to small bowel obstruction. Hernia repairs were performed through open (n=25), robotic (10), and laparoscopic approaches (1). Surgical techniques included direct repair with mesh (20), direct repair without mesh (4), stoma relocation with mesh (5), and stomarelocation without mesh (7). The 90-day complication rate was 28%. In a median follow-up of 24 (7–47) months, 17 patients (47%) had a recurrence. The median time to recurrence was 9 (7–24) months. On multivariable analysis, 90-day complication following PSH repair was associated with an increased risk of recurrence.

**Conclusions:**

In this report of one of the largest series of PSH repair in the Urology literature, 47% of patients had a recurrence following hernia repair with a median follow-up time of 2 years. There was no significant difference in recurrence rates when comparing repair technique or the use of open or minimally invasive approaches.

**Supplementary Information:**

The online version contains supplementary material available at 10.1007/s00345-024-05123-w.

## Introduction

ladder cancer is one of the most common malignancies worldwide [1]. For muscle-invasive bladder cancer as well as high-risk non-invasive variants, radical cystectomy (RC) with urinary diversion is the ‘gold-standard’ treatment [2]. While the choice of urinary diversion is individualized, ileal conduits (IC) represent the fastest, easiest, least complication-prone, and most commonly performed urinary diversion [3]. Recent trends in urinary diversion after RC show an increasing rate of IC, especially at high-volume centers that perform the majority of RCs in a minimally invasive fashion [4]. Studies have also confirmed most patients in the US have an IC following bladder cancer surgery [5].

Parastomal hernia (PSH), the protrusion of peritoneal contents through the abdominal wall defect adjacent to the stoma, is one of the most common complications of IC [6]. Previous work by our group showed that up to 30% of patients had radiological evidence of PSH following RC with IC [6]. Multiple studies have investigated risk factors for PSH, and most agree that obesity and female gender are associated with an increased risk of PSH [7–10].

While most PSHs are asymptomatic, problems can arise, ranging from mild discomfort to life-threatening complications, such as perforation, obstruction, and strangulation [11]. Our previous work showed 25% of PSH had evidence of progression, with a median of 12 months [7]. Most PSHs can be managed nonoperatively. Surgical management is typically reserved for patients who have intractable issues, including skin irritation or difficulty with pouching the ostomy, or if life-threatening complications occur [11].

Management options for PSH include direct fascial repair, mesh repair, and stoma relocation [12]. These options all have the potential for recurrence following primary PSH repair [13, 14]. However, there is a paucity of data in the urology literature regarding the outcomes of different PSH repair techniques and incidence of recurrent PSH. [15]. In this study, we aim to report perioperative and long-term postoperative outcomes of patients with IC urinary diversion undergoing PSH repair.

## Methods

### Study population

In this single-center retrospective study, using our institutional review board-approved cystectomy database (IRB# HS-01B014), we reviewed records of patients who underwent PSH repair following cystectomy and IC urinary diversion between 2003 and 2022. Patients with unavailable/insufficient follow-up data were excluded.

### Data collection and outcome measures

Baseline clinical and pathological variables included age, sex, chronic obstructive pulmonary disease (COPD), diabetes mellitus, body mass index (BMI), history of abdominal surgery, presence of concomitant hernia, indication for PSH repair, etiology of cystectomy, cystectomy-PSH repair interval, usage of prophylactic mesh at the time of IC construction, surgical approach of cystectomy and PSH, PSH repair technique (direct repair vs. stoma relocation with or without mesh), length of hospital stay, and 90-day complications. For patients with PSH recurrence, time to recurrence and details of secondary PSH repair were recorded.

The primary outcome was clinical hernia recurrence. Secondary outcomes included perioperative complications of primary PSH repair, rate of recurrence based on PSH repair techniques, risk factors for hernia recurrence, and complications related to secondary PSH repair.

### Statistical analysis

Demographic and clinical features were summarized and analyzed using Chi-squared and Wilcoxon tests for categorical and continuous variables, respectively. Univariate and multivariable Cox regression analyses were performed to test for the associations between different variables and PSH recurrence.

The statistical software package IBM SPSS (Version 28) was used for all the analyses in this study. All *P* values reported were two-sided and *P* values < 0.05 were taken to indicate statistical significance.

## Results

Thirty-six patients (19 females,17 males) with a median (IQR) age of 79 (73–82) years were included. Cystectomies were performed for bladder cancer in 30 and for benign etiologies in 6 patients. The median time between cystectomy and PSH repair was 29.8 (14.4–49) months. The baseline features of the patients are presented in Table 1.

**Table 1:** Demographics and baseline features of patients

Variables	Value
Age, median (IQR), years	80 (72–82)
Gender, n (%) male female	17 (47%) 19 (53%)
BMI, median (IQR)	28 (25–31)
DM, n (%)	10 (28)
COPD, n (%)	5 (14)
Concomitant hernia, n (%)	17 (47)
RCx indication, n (%) Cancer Benign	6 (17) 30 (83)
Mesh at initial RCx, n (%)	3 (8)
RCx approach, n (%) Open MIS	25 (69) 11 (31)
PSH repair indication, n (%) Elective SBO	32 (89) 4 (11)
PSH repair approach, n (%) Open MIS	25 (69) 11 (31)
PSH repair technique, n (%) DR with mesh DR without mesh RL with mesh RL without mesh	20 (56) 4 (11) 5 (14) 7 (19)

BMI: body mass index; DM: diabetes mellitus; COPD: chronic obstructive pulmonary disease; RCx: radical cystectomy; MIS: minimally invasive surgery; SBO: small bowel obstruction; DR: direct repair; RL: relocation

### Surgical data

Most PSH repairs were performed due to elective concerns, such as abdominal discomfort and/or ostomy appliance issues (*n* = 32); 4 patients required surgery due to small bowel obstruction. Hernia repairs were performed through open, robotic, and laparoscopic approaches in 25, 10, and 1 patient(s), respectively. None of the minimally-invasive PSH repairs were converted to open. Surgical techniques included direct repair with mesh (*n* = 20), direct repair without mesh (*n* = 4), stoma relocation with mesh (*n* = 5), and stoma relocation without mesh (*n* = 7).

### Perioperative outcomes

There was one intraoperative complication (enterotomy) that was repaired successfully. The median (IQR) length of stay was 4 (2–7) days. The 90-day complication rate was 28% (10/36), including 11% (4/36) high-grade and 17% (6/36) low-grade (Table 2).

**Table 2:** 90-day complications of parastomal hernia repair

Complication type (*n*)*	Grade^#^	Management
Ileus (2)	1	Conservative
Aspiration pneumonia (1)	2	Medical therapy
Congestive Heart Failure Exacerbation (1)	2	Medical therapy
Fever (1)	2	Medical therapy
Pulmonary Embolus (1)	2	Medical therapy
UTI and hydronephrosis (1)	3a	Nephrostomy placement
SBO and abdominal wall abscess (1)	3b	Surgery
SSI and wound dehiscence (1)	3b	Surgery
Sepsis (1)	4	Medical therapy with ICU admission

#Clavien-Dindo classification

UTI: urinary tract infection; SBO: small bowel obstruction; SSI: surgical site infection; ICU: intensive care unit

### Long-term outcomes

**Fig. 1:**
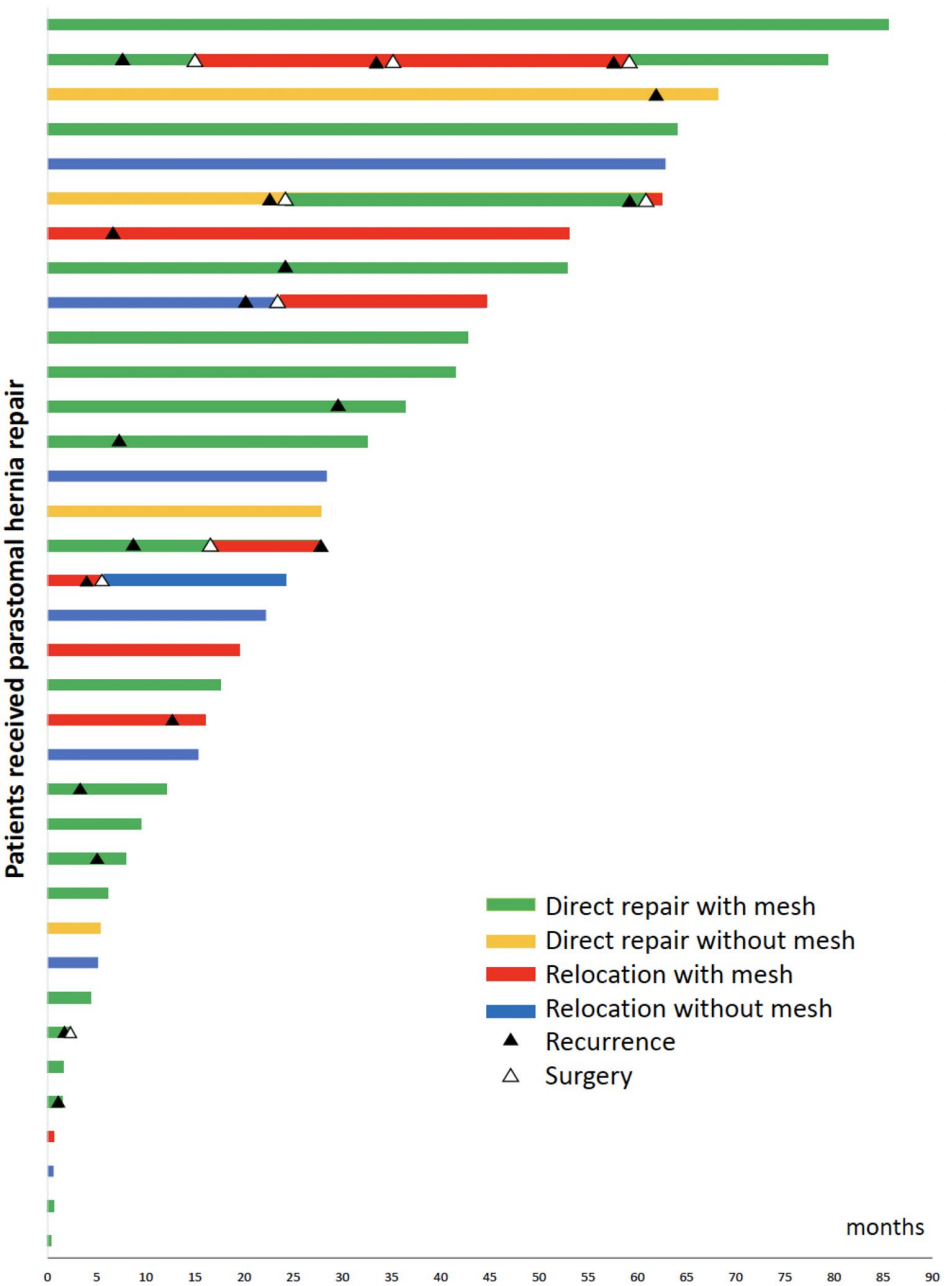
Swimmer’s plot demonstrating the outcomes of patients undergoing parastomal hernia repair using different techniques (each bar represents one patient). (COLOR)

With a median (IQR) follow-up of 24.3 (7.3–46.7) months after PSH repair, 17 (47%) patients had a recurrence. The median (IQR) time to recurrence was 8.6 (6.8–24) months. Recurrence rates for direct repair and stoma relocation techniques were 54% (13/24) and 33% (4/12), respectively (HR 0.59, *P* = 0.26). There was no difference in robotic/lap vs. open approaches in terms of hernia recurrence (HR 0.47, *P* = 0.14). Six cases underwent PSH repair for recurrent hernia, of whom three patients developed re-recurrence requiring surgery (Fig. 1). Cox regression analysis showed patients experiencing perioperative complications were more prone to develop hernia recurrence (HR 3.5, 95% CI 3.5 (1.12–10.98); *P* = 0.032). The PSH repair technique for a recurrent PSH (direct repair vs. stoma relocation) did not show a statistically significant difference on multivariable regression (HR 0.78, 95% CI 0.29–2.11; *P* = 0.62) (Table 3).

**Table 3:** Univariate and multivariable cox regression analyses of factors affecting PSH recurrence

Variables	Univariate	Multivariable
	HR (95% CI), *P* value	HR (95% CI), *P* value
Age (cont.)	0.99 (0.93–1.06), 0.78	
Gender, male vs. female	0.93 (0.36–2.37), 0.87	
BMI (cont.)	1.02 (0.93–1.12), 0.68	
DM	0.78 (0.28–2.21), 0.64	
COPD	1.32 (0.38–4.59), 0.66	
Concomitant hernia	1.4 (0.55–3.56), 0.48	
RCx indication, cancer vs. benign	0.47 (0.13–1.69), 0.25	
Mesh at initial RCx	0.76 (0.1–5.79), 0.79	
RCx approach, open vs. MIS	0.58 (0.22–1.52), 0.27	
PSH repair indication, SBO vs. elective	1.58 (0.35–7.17), 0.56	
PSH repair approach, open vs. MIS	0.47 (0.17–1.29), 0.14	
PSH repair technique, DR vs. RL	0.59 (0.23–1.49), 0.26	0.78 (0.29–2.11), 0.62
PSH repair with mesh	0.82 (0.32–2.09), 0.67	
90-day complications	3.84 (1.29–11.39), 0.015	3.5 (1.12–10.98, 0.032

BMI: body mass index; DM: diabetes mellitus; COPD: chronic obstructive pulmonary disease; RCx: radical cystectomy; MIS: minimally invasive surgery; SBO: small bowel obstruction; DR: direct repair; RL: relocation; cont.: continuouss

## Discussion

This is one of the largest series of patients undergoing PSH repair following cystectomy and IC with long-term follow-up following hernia repair, with either open or robotic techniques. Our results demonstrate that a PSH involving an IC is challenging and repair of PSH has a high recurrence rate.

Our group’s previous work highlighted a 30% incidence of PSH following RC at our institution, with patient factors including female gender, diabetes, chronic obstructive pulmonary disease and higher body mass index as independent risk factors [7]. In this study, we highlighted that the most common indication for PSH repair was elective; however, the remainder were indicated due to small bowel obstruction. Review of the available literature shows most PSH repairs are elective, with reports of emergent repair due to obstruction and/or strangulation ranging from 2 to 15% [9, 10]. A Danish nationwide study among colostomy patients found emergent PSH repair was the strongest risk factor for reoperation or death [16].

Stoma-related complications have been reported at rates of up to 60%, with reported rates varying widely [7, 17, 18]. Patients undergoing urgent surgeries are associated with a higher rate of complications [16]. In our series, 28% of patients had complications within 90 days of primary PSH repair, most of which were infection or wound related. In a Finnish nationwide cohort study of 235 patients, the most common complications within 30 days of primary PSH repair were infectious (15%) or bleeding related (4%); on extended follow-up, small bowel obstruction was present in 9% of the cohort [19].

Our series found 47% of patients had a PSH recurrence, which is in line with previous studies. A meta-analysis noted 3 studies reporting recurrences between 27 and 50% [18]. A series of 28 patients reported a lower recurrence rate of 18% [19]. A recent 51-patient series from France quoted a recurrence rate of 35% [20]. Interestingly, the French series had a higher rate of recurrence seen in a short-term follow-up window, indicating the time to PSH recurrence may be variable.

One-third of patients with a PSH recurrence in our series underwent repeated PSH, higher than other reports in the literature. Mäkäräinen-Uhlbäck et al. reported a re-operation rate of 14% in their 28-patient series, with a median follow-up of 30 months [19]. An older 95-patient series of colostomy patients showed that 18% underwent recurrent PSH repair [21]. A more recent colostomy PSH series by Näsvall et al. highlighted a reoperation rate of 24% within 12 months of repair [22].

We found no significant difference in PSH recurrence rates when considering a patient’s age, sex, BMI, presence of diabetes, presence of COPD, concomitant hernia, or indication for initial cystectomy. There is a lack of consensus on what factors may predispose patients to a PSH; nevertheless, some studies indicate factors like BMI, sex, age, chronic respiratory disorders, and malnutrition [7, 8, 23]. A significant difference was seen in PSH recurrence for patients who had peri-operative complications at time of repair (HR 3.84). Further research, with longer follow-up is warranted to better understand predisposing factors for PSH recurrence.

Direct repair patients had a recurrence rate of 54% compared to 33% in patients with relocation, although this difference was not statistically significant. Of all repair techniques, direct repair is the least complicated. Typically, it involves reduction of the hernia, excision of the hernia sac and attenuated scar tissue, and the re-approximation of healthy fascia with suture [24]. Although there are some advantages of direct fascial repair such as technique simplicity and maintenance of current stoma position, overall results are poor [12]. Previous studies have indicated direct repair with fascial tissue had recurrence rates of up to 76% [25].

The other mainstay for PSH repair is relocation, which can be useful when current stoma position is unsatisfactory. When considering the colorectal literature, Riansuwan et al. report that relocation on the same side of the abdomen had as high recurrence rates similar to direct repair, while contralateral relocation had a significantly lower recurrence rate [12]. Our series found no significant difference in recurrence between the two methods. This could be due to the medium-term follow-up period of our series or the sample size. Additionally, relocation poses its own challenges due to ureteric anastomoses, short mesentery of the ileal conduit [15], and risk of hernia recurrence at either site in the future.

Our study did not show a difference between PSH repairs with or without mesh; however, previous series highlighted a lower recurrence rate with mesh [26]. As patients with longer follow-up times accrue, special attention must be paid to the reporting of mesh-specific complications, such as mesh infection, adhesions, and erosion. Investigators are also querying whether prophylactic mesh at the time of IC creation will influence PSH rates. One randomized controlled trial found no significant difference in development of a clinical PSH within 2 years but did show a difference after 3 years [27]. This was similar to the initial findings of the trial performed by our group [28].

Our series showed no difference in PSH recurrence rates between open (47%) and minimally invasive (48%) approaches for initial PSH repair. There is limited data comparing minimally invasive and open PSH repair techniques. Around the introduction of the robotic PSH approach, open repair recurrence rates were quoted around 30% [29]. Robotic approaches have been said to provide multiple benefits, such as enabling adhesiolysis of small bowel, avoiding penetrative fixation techniques and easy suture closure of the hernia defect [30]. However, the robotic approach can have its own difficulties, such as difficult dissection of firmly adherent hernia sac and lack of widespread adoption.

Given the high rate of PSH following IC creation, and that series with shorter follow-up may not capture the full nature of PSH recurrence patterns [16], characterizing the long-term results of PSH repair is critical to informing how to optimize patients’ quality of life. Our study captured multiple repair techniques, including direct repair and relocation, both with and without mesh. Further, we report on cases that include both open and minimally invasive approaches. There are a few limitations to this study. This is a retrospective review without randomization. This may lead to selection biases. Furthermore, there may have been selection bias in the approach, type of repair, or use of mesh depending on patient clinical factors or aspects, such as the hernia size. Additionally, this was a single-institution study at a high-volume surgical center, and results may not be widely generalizable. Furthermore, this study was carried out at a tertiary referral center, and some patients may have chosen to follow-up more locally. We also do not provide technical details such as hernia size, specific repair steps, or type of mesh, which can all vary and impact outcomes. A greater number of patients with long follow-up may be required to sufficiently power some of the questions at hand.

## Conclusion

In this report, nearly half of patients experienced a PSH recurrence following hernia repair with a median follow-up time of 2 years. There was no significant difference in recurrence rates when comparing repair techniques and surgical approaches. In addition, 90-days complication following PSH repair was associated with an increased risk of hernia recurrence.

## Electronic supplementary material

Below is the link to the electronic supplementary material.

Supplementary Material 1
