# Supplementary material for: Perioperative and extended outcomes of patients undergoing parastomal hernia repair following cystectomy and ileal conduit
Source: World J Urol. 2024 Aug 12;42(1):482. doi: 10.1007/s00345-024-05123-w (PMC11319381; doi:10.1007/s00345-024-05123-w)
Supplement: Supplementary file 1 — Supplementary Material 1 [file 345_2024_5123_MOESM1_ESM.docx]

**Perioperative and Extended Outcomes of Patients Undergoing Parastomal Hernia Repair Following Cystectomy and Ileal Conduit**

Taseen F. Haque^a^, Alireza Ghoreifi^a^, Farshad Sheybaee Moghaddam^a^, Masatomo Kaneko^a^, David Ginsberg^a^, Rene Sotelo^a^, Inderbir Gill^a^, Mihir Desai^a^, Monish Aron^a^, Anne Schuckman^a^, Siamak Daneshmand^a^, Hooman Djaladat^a*^

^a^USC Institute of Urology, Catherine and Joseph Aresty Department of Urology, University of Southern California, Los Angeles, CA, USA.

* Corresponding Author:

Hooman Djaladat, M.D., M.S.

USC Institute of Urology and Catherine and Joseph Aresty Department of Urology

Address: 1441 Eastlake Ave, Suite 7416, Los Angeles, CA 90089

Tel: +1-323-865-3700, Fax: + 1-323-865-0120

Email: djaladat@med.usc.edu

**Word Count:** Abstract (248); Text (1999+(250x4)=2999)

**Keywords (4-6):** Abdominal Hernia; Cystectomy; Herniorrhaphy; Surgical Mesh; Urinary Diversion

**Competing Interests/Declaration of Interests:** All authors have no conflict of interest related to this research to declare.

**Financial disclosures:** This research received no specific grant from any funding agency in the public, commercial, or not-for-profit sectors.

**Abstract**

**PURPOSE:** To report perioperative and long-term postoperative outcomes of cystectomy patients with ileal conduit (IC) urinary diversion undergoing parastomal hernia (PSH) repair.

**METHODS:** We reviewed patients who underwent cystectomy and IC diversion between 2003 and 2022 in our center. Baseline variables, including surgical approach of PSH repair and repair technique, were captured. Multivariable Cox regression analysis was performed to test for the associations between different variables and PSH recurrence.

**RESULTS:** Thirty-six patients with a median (IQR) age of 79 (73–82) years were included. The median time between cystectomy and PSH repair was 30 (14–49) months. Most PSH repairs (32/36, 89%) were performed electively, while 4 were due to small bowel obstruction. Hernia repairs were performed through open (n=25), robotic (10), and laparoscopic approaches (1). Surgical techniques included direct repair with mesh (20), direct repair without mesh (4), stoma relocation with mesh (5), and stoma relocation without mesh (7). The 90-day complication rate was 28%. In a median follow-up of 24 (7–47) months, 17 patients (47%) had a recurrence. The median time to recurrence was 9 (7–24) months. On multivariable analysis, 90-day complication following PSH repair was associated with an increased risk of recurrence.

**CONCLUSIONS:** In this report of one of the largest series of PSH repair in the Urology literature, 47% of patients had a recurrence following hernia repair with a median follow-up time of 2 years. There was no significant difference in recurrence rates when comparing repair technique or the use of open or minimally invasive approaches.
